# Evaluation of shade correspondence between high-translucency pre-colored zirconia and shade tab by considering the influence of cement shade and substrate materials

**DOI:** 10.1016/j.heliyon.2023.e23046

**Published:** 2023-11-29

**Authors:** Chien-Ming Kang, Yen-Wen Huang, Sheng-Han Wu, Yuichi Mine, I-Ta Lee, Tzu-Yu Peng

**Affiliations:** a Huayi Dental Laboratory, Taipei, Taiwan; b School of Dentistry, College of Oral Medicine, Taipei Medical University, Taipei, Taiwan; c Department of Dentistry, Wan Fang Hospital, Taipei Medical University, Taipei, Taiwan; d Department of Medical System Engineering, Graduate School of Biomedical and Health Sciences, Hiroshima University, Hiroshima, Japan

**Keywords:** Monolithic multilayer zirconia, CAD-CAM, Colorimetry, Color, Resin cements, Dental abutments

## Abstract

**Statement of problem:**

The relation between cement color and abutment substrate material and the corresponding effect on the color accuracy of high-transparency pre-colored zirconia (HT-Zr) remains unclear.

**Purpose:**

This in-vitro study aimed to investigate the difference in color accuracy when the HT-Zr is bonded to different materials-based substrates with differently colored resin cement.

**Materials and methods:**

Vita A1 shade HT-Zr with 1 mm thickness was used as the testing sample. The samples were first placed on zirconia (ZR), tooth color resin (CR), and metallic (MT) abutment substrates. Subsequently, four differently colored cements (translucent (TR), bleach, opaque, and A2 shade (A2)) were used for bonding HT-Zr onto the substrate, and the non-bonded group was used as the control group (CG). There were 15 groups in total (n = 10 per group). A digital colorimeter was used to obtain Commission Internationale de l’Eclairage (CIELab) color parameters. The translucency parameter (TP_00_) of the substrate and sample, as well as color difference (ΔE_00_) and chroma (C) between the different groups were calculated. Additionally, the ΔE_00_ and TP_00_ were compared with the moderately unacceptable match of ΔE_00_ = 3.6. The statistical analysis was conducted using ANOVA and Tukey HSD post-hoc test (α = 0.05).

**Results:**

HT-Zr exhibited high translucency (TP_00_ = 11.02 ± 0.18), and the mean ΔE_00_ of the testing samples ranged between 2.18 ± 0.20 and 13.14 ± 0.31. The ZR-CG and MT-A2 groups showed the highest and lowest lightness separately. The CR-CG group exhibited the highest C, and the ΔE_00_ was lower than that of 3.6. The MT-TR group showed the lowest C and the highest ΔE_00_. The inter-group comparison revealed that the ΔE_00_ for different cement is mostly lower than the acceptable color match of 1.0; moreover, the ΔE_00_ for all the substrates, excluding the CG group, is higher than 3.6.

**Conclusions:**

The abutment substrate materials and the cement color should be considered with caution when using HT-Zr, with the effect of abutment substrate materials being more apparent in color accuracy. HT-Zr restorations are not recommended for discolored or bleached abutments but only for natural-colored abutments to achieve the optimal color appearance.

**Clinical Implications:** When using high-translucency monolithic pre-colored zirconia, the suitability of the abutment substrate materials must be considered in addition to the color of the cement to decrease color distortion and increase color accuracy for achieving the expected aesthetic performance.

## 1 Introduction

Aesthetics are highly valued in current society, and our teeth considerably affect our appearance [1,2]. Porcelain fused to zirconia (PFZ) can achieve good optical properties through veneering porcelain to match the color of natural teeth [3]; however, there is risk of chipping [4,5]. The veneer ceramic may not be strong enough to handle occlusal stresses, the coping and veneering ceramics may have different thermal expansion coefficients, and the veneering may not be wettable enough. To circumvent all the concerns, monolithic zirconia has been developed [3,[6], [7], [8], [9]]. Monolithic zirconia, which does not require veneering ceramic and enables attaining the aesthetic requirements in anterior teeth only through digital processes and simple staining steps, has gradually attracted considerable interest from prosthodontists [10,11]. A previous study used natural teeth as benchmarks to manufacture PFZ and monolithic zirconia restorations, and examined their optical characteristics. The results demonstrated negligible differences. Moreover, another study reported that the monolithic zirconia eliminates the risk of chipping [12,13].

Recently, high-translucency monolithic multilayer pre-colored zirconia (HT-Zr) has been developed [[14], [15], [16]]. Its advantages include high translucency and the possibility of having a gradient color [[17], [18], [19]]. The restorations produced from it thus have a color similar to that of natural teeth, which significantly improves its clinical applicability. However, there still are difficulties in achieving the ideal color accuracy using zirconia restorations, which can only be attained if the colors of the restoration, abutment, and cement match each other [20].

Paravina et al. emphasized that visual thresholds in dentistry determine color, translucency, and whiteness match/mismatch, playing a crucial role in quality control, material evaluation, clinical performance assessment, research analysis, and standardization [21]. Literature suggests that an acceptable color match ranges (ΔE_00_) from 0.8 to 1.0, while moderately unacceptable color match have values (ΔE_00_) lower than 3.6 [[21], [22], [23], [24]]. Besides, the higher the HT-Zr thickness, the lower is the translucency and the better the masking ability; however, low translucency also results in high color difference (ΔE_00_) and non-ideal color. Prior studies have concluded that 1-mm-thick HT-Zr is the most suitable thickness that can simultaneously meet aesthetic and strength requirements [18] and also inferred that the effects of color accuracy are lower when the substrate color is close to that of multilayer zirconia [19].

Dental cements secure prosthetic components to prepared teeth or dental implants, influencing periodontal and mucosa health, sealing against microleakage, and enhancing long-term stability [25]. Importantly, dental cement also acts as a masking element, capable of hide a colored background and influencing the aesthetic aspect of restoration [26]. Some literature reports reviewed the masking ability and optical properties of cement on monolithic or bilayer ceramic restorative systems [[27], [28], [29], [30]]; however, only few studies examine the effects of cement shade on the color accuracy of HT-Zr [30,31]. Herein, this study aims to investigate the effects of different cement shade and abutment substrate materials on the color accuracy of HT-Zr. These experimental results will establish the matching of abutment material and cement shade to achieve the expected optical performance so as to make future clinical operations more convenient. The null hypothesis tested was that the target shade would not be altered by cement shade and substrate material.

## 2 Materials and methods

Three substrates (Table 1), including high translucency zirconia (ZR), tooth color resin (CR), and metal (MT), were used in this study to simulate different abutment materials. The 10 × 10 mm substrate plates with a thickness of 2.0 mm were designed using 3D model software. The ZR was processed using a dental milling machine (Cameo 250i, Aidite Technology Co., Ltd., Qinghuangdao, Mainland China). The CR and MT were 3D printed by Phrozen Sonic XL 4 K (Phrozen Tech Co., Ltd., Hsinchu, Taiwan) and Riton Laser D-100 (Rxton Technology Co., Ltd., Guangdong, Mainland China), respectively. The Vita A1 shade HT-Zr test piece with dimensions of 8 × 8 mm with a thickness of 1.0 mm was prepared using a dental CAD-CAM system. The sample size of n = 10 for each group was calculated based on a power analysis using SPSS software (IBM SPSS Statistics v19.0; IBM Corp., Armonk, NY, USA) with 80 % power and 0.05 level of significance, which enabled clinically justified recommendations. Finally, all the specimens were ultrasonically cleaned, air-dried, and were not subjected to any staining or polishing.

**Table 1:** Materials used in this study and their properties.

Materials/Trade Name		Main Composition^a^	Manufacturer	Abbr.
***High-transparency pre-colored zirconia*** 3D Pro Zir (Vita A1 shade)		Y_2_O_3_+ ZrO_2_ (4 YPSZ+5YPSZ)	Aidite Technology Co., PR China	HT-Zr
***Zirconia substrate*** Superfect Zir		Y_2_O_3_+ ZrO_2_ (3Y-TZP)	Aidite Technology Co., PR China	ZR
***Tooth color resin substrate*** NextDent C&B MFH (N1 shade)		methacrylic oligomers, phosphine oxide, microfiller	NextDent B.V, The Netherlands	CR
***Metallic substrate*** C02 CoCrMo Powders		Co, Cr, Mo	Material Technology Innovations Co., PR China	MT
***Resin cement*** G-CEM LinkForce	Translucent	Paste A: Bis-GMA, UDMA, dimethacrylate, etc. Paste B: Bis-MEPP, UDMA, dimethacrylate, etc.	GC Corp., Japan	TR
	Bleach			BL
	Opaque			OP
	A2 shade			A2

a According to the information provided by the manufacturer. 4Y-PSZ, 4 mol% yttria-partially stabilized zirconia; 5Y-PSZ, 5 mol% yttria-partially stabilized zirconia; 3Y-TZP, 3 mol% yttria-partially stabilized tetragonal zirconia polycrystal; Bis-GMA, bisphenol-A-glycidyl methacrylate; UDMA, urethane dimethacrylate; Bis-MEPP, bisphenole-A-ethoxylate dimethacrylate.

**Fig. 1:**
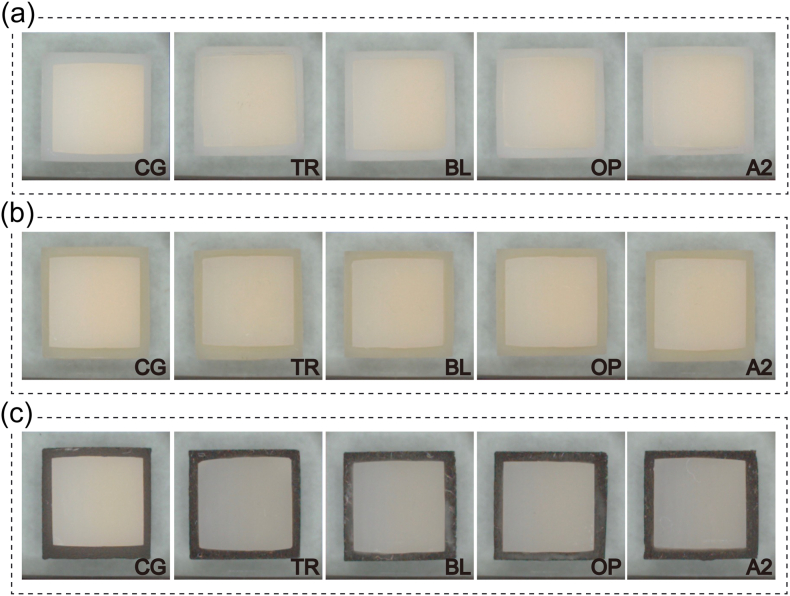
Precolored HT-Zr samples. The HT-Zr samples were placed on ZR (a), CR (b), and MT (c) substrates. In the case of CG, cement bonding was not used, while in the other cases, samples were bonded with four different shades of cement (TR, BL, OP, and A2), as indicated in the lower right corner of the respective graphics.

The resin cement was selected from 4 different shades (Table 1): transparent (TR), bleach (BL), opaque (OP), and A2 shade (A2). Cement was not used in the control group (CG). A layer of resin cement (G-CEM LinkForce; GC Corp, Tokyo, Japan) was applied onto the substrate, and HT-Zr was placed on top of it and pressed under a constant load of 4.9 N for 5 min to ensure uniform thickness. Subsequently, the excess cement was removed and the resultant sample was light-cured twice for 10s using a light curing machine (Litex 696; Dentamerica, CA, USA) (Fig. 1).

**Fig. 2:**
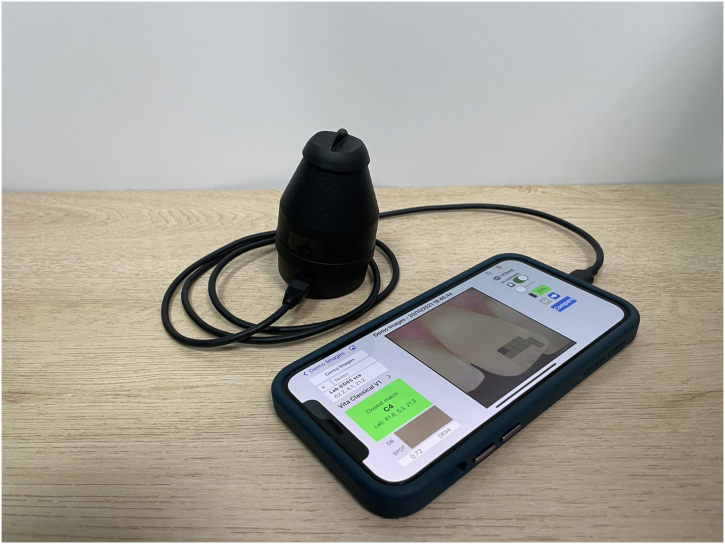
Digital colorimeter.

The color of the test samples was measured using a digital colorimeter (OptiShade StyleItaliano, Smile Line, St-Imier, Switzerland) (Fig. 2) to quantify the color parameter - lightness (L*), red-green coordinates (a*), yellow-blue coordinates (b*) - stipulated by the International Commission on Illumination Systems (CIELab). The colorimeter, accompanied by a smartphone application (OptiShade App iOS, StyleItaliano, Smile Line, St-Imier, Switzerland) and a shade-matching software program (Matisse Software, LabMatisse, Smile Line, St-Imier, Switzerland), can predict the shade of the samples effectively without using traditional shade guides [32]. A total of 15 color parameter (L*, a*, b*) values were acquired from every group, excluding values with large errors, and the final color parameters were obtained as averages of n = 10. In addition, the non-bonded substrate and test sample were placed on the black (L* = 22.3, a* = -0.5, and b* = -0.9) and white (L* = 90.9, a* = -0.7, and b* = 1.8) calibration cards (QP Card 101; QPcard AB, Helsingborg, Sweden) for color measurement (n = 10 per group). The following formula was used to calculate the translucency parameter (TP_00_) explaining the masking ability of the material, ${\text{TP}}_{00}={\left({\left(\frac{L_{B}^{\prime }‐L_{W}^{\prime }}{k_{L}{\;S}_{L}}\right)}^{2}+{\left(\frac{C_{B}^{\prime }‐C_{W}^{\prime }}{k_{C}{\;S}_{C}}\right)}^{2}+{\left(\frac{H_{B}^{\prime }‐H_{W}^{\prime }}{k_{H}{\;S}_{H}}\right)}^{2}+R_{T}\left(\frac{C_{B}^{\prime }‐C_{W}^{\prime }}{k_{C}{\;S}_{C}}\right)\left(\frac{H_{B}^{\prime }‐H_{W}^{\prime }}{k_{H}{\;S}_{H}}\right)\right)}^{\frac{1}{2}}$, where the L’_B_, C’_B_, and H’_B_, respectively, represent the lightness, chroma, and hue of the sample on a black background, and L’_W_, C’_W_, and H’_W_ represent the lightness, chroma, and hue, respectively, of the sample on a white background; k_L_, k_C_, and k_H_ are the weights for lightness, chroma, and hue, respectively; S_L_, S_C_, S_H_ are the mean coefficients for lightness, chroma, and hue, respectively; R_T_ is the overall calibration coefficient based on chroma and hue differences.

Chroma (C), $C={\left({\left(a^{*}\right)}^{2}+{\left(b^{*}\right)}^{2}\right)}^{\frac{1}{2}}$ is used to determine color purity and saturation. Concerning ΔE_00_, this study measured (1) color parameters of HT-Zr for different cement and substrates, and ΔE_00_ between HT-Zr and Vita A1 shade guide (L* = 76.7, a* = 1.1, b* = 14.7); (2) ΔE_00_ of HT-Zr when using the same substrate but different cement; (3) ΔE_00_ of HT-Zr when using different substrates but same cement. The following equation was used for calculation: ${\Delta E}_{00}={\left({\left(\frac{\Delta L’}{k_{L}{\;S}_{L}}\right)}^{2}+{\left(\frac{\Delta C’}{k_{C}{\;S}_{C}}\right)}^{2}+{\left(\frac{\Delta H’}{k_{H}{\;S}_{H}}\right)}^{2}+R_{T}\left(\frac{\Delta C’}{k_{C}{\;S}_{C}}\right)\left(\frac{\Delta H’}{k_{H}{\;S}_{H}}\right)\right)}^{\frac{1}{2}}$, where the ΔL’, ΔC’, and ΔH’ represent lightness, chroma, and hue differences, respectively.

Shapiro–Wilk and Levene's tests were used to analyze the normality and homogeneity of the measured values. As the data appeared homogeneous and normally distributed, the parental analysis was performed. One-way ANOVA was used to analyze the color parameter differences between the test samples and Vita A1 shade guide. Subsequently, two-way ANOVA was used to analyze the effects of cement shade and substrate materials on C and ΔE_00_. Finally, the Tukey HSD post-hoc test was used to examine the differences between the groups. All statistical analyses were performed by using SPSS software (α = 0.05).

## 3 Results

Table 2 shows the color parameters (L*, a*, b*) of 15 combinations of cement and substrates. The results indicate marginal differences between the L* of Vita A1 and those of ZR-TR (P = 0.67), ZR-OP (P = 0.36), ZR-A2 (P = 0.49), and CR-CG (P = 0.99). Additionally, HT-Zr has a high TP_00_ of 11.02 ± 0.18; among the three tested substrates, CR has the highest TP_00_ (7.71 ± 0.13), followed by ZR (7.09 ± 0.07), and MT acquiescence has zero TP_00_.

**Table 2:** CIELab color attributes (Mean ± standard deviation) of each group.

Group		L*	a*	b*
Vita A1 shade guide		76.70	1.1	14.7
ZR	CG	79.59 ± 0.17^※^	0.14 ± 0.09^※^	9.73 ± 0.31^※^
	TR	77.03 ± 0.19	−0.45 ± 0.19^※^	8.62 ± 0.29^※^
	BL	78.06 ± 0.22^※^	−1.46 ± 0.11^※^	8.49 ± 0.37^※^
	OP	77.09 ± 0.11	−0.53 ± 0.25^※^	8.63 ± 0.29^※^
	A2	77.06 ± 0.57	−1.32 ± 0.14^※^	8.43 ± 0.34^※^
CR	CG	76.54 ± 0.30	−0.01 ± 0.11^※^	12.00 ± 0.26^※^
	TR	73.35 ± 0.29^※^	−0.73 ± 0.15^※^	11.87 ± 0.24^※^
	BL	74.52 ± 0.38^※^	−0.93 ± 0.20^※^	11.84 ± 0.17^※^
	OP	73.57 ± 0.27^※^	−0.48 ± 0.15^※^	11.69 ± 0.16^※^
	A2	73.48 ± 0.49^※^	−0.64 ± 0.17^※^	11.61 ± 0.12^※^
MT	CG	72.92 ± 0.24^※^	0.08 ± 0.18^※^	8.24 ± 0.25^※^
	TR	63.63 ± 0.31^※^	−1.20 ± 0.08^※^	3.76 ± 0.32^※^
	BL	65.46 ± 0.21^※^	−1.35 ± 0.09^※^	4.15 ± 0.23^※^
	OP	65.27 ± 0.43^※^	−1.21 ± 0.09^※^	4.44 ± 0.25^※^
	A2	63.63 ± 0.48^※^	−1.07 ± 0.12^※^	3.93 ± 0.29^※^

The CIELab color values of the Vita A1 shade guide were derived from the database of the digital colorimeter (Optihue Styleitaliano; Smile Line SA); the mark (※) indicates the values that significantly differ from the Vita A1 shade guide based on Student's t-test (P < 0.05).

**Fig. 3:**
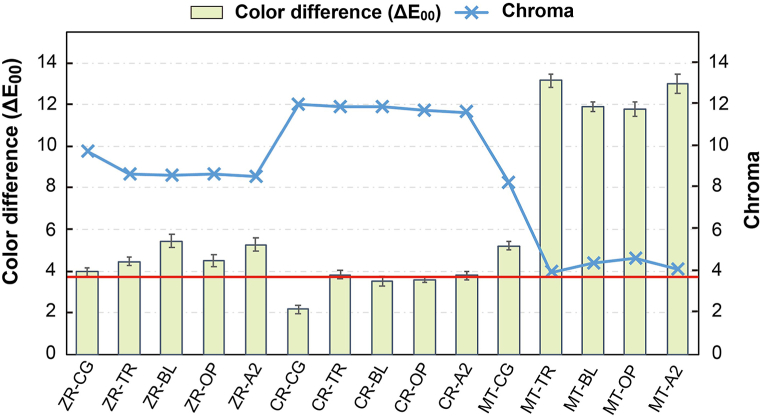
Color differences (ΔE_00_) and chroma values for different groups. Red line represents a moderately unacceptable color match of 3.6.

The C values of different groups as well as ΔE_00_ between the color parameters of Vita A1 shade guide and those of the experimental groups are shown in Fig. 3 and Table 3. The results indicate that the cement shade did not significantly affect the C value when the same substrate was used (P > 0.05). The ΔE_00_ of CR-CG (2.18 ± 0.20), CR-BL (3.54 ± 0.23), and CR-OP (3.58 ± 0.13) are lower than moderately unacceptable color match (ΔE_00_ = 3.6), whereas the ΔE_00_ of CR-TR (3.83 ± 0.19), CR-A2 (3.78 ± 0.22), and ZR-CG (3.96 ± 0.22) are slightly higher than moderately unacceptable color match. Notably, all the ΔE_00_ of the ZR and MT groups are greater than moderately unacceptable color match, among which MT-TR (13.14 ± 0.31) shows the highest ΔE_00_.

**Table 3:** Results of 2-way ANOVA on effects of cement color and substrate materials on color difference (ΔE_00_) and chroma (C).

Source	Type III Sum of Squares	df	Mean Square	F	P
ΔE_00_					
Cement color	259.40	4.00	64.85	941.59	<.001
Substrate materials	1652.47	2.00	826.24	11996.58	<.001
Cement color × Substrate materials	208.12	8.00	26.01	377.72	<.001
Error	9.30	135.00	0.07		
C value					
Cement color	77.22	4.00	19.3	281.74	<.001
Substrate materials	1151.88	2.00	575.94	8405.73	<.001
Cement color × Substrate materials	64.33	8.00	8.04	117.36	<.001
Error	9.25	135.00	0.07		

**Fig. 4:**
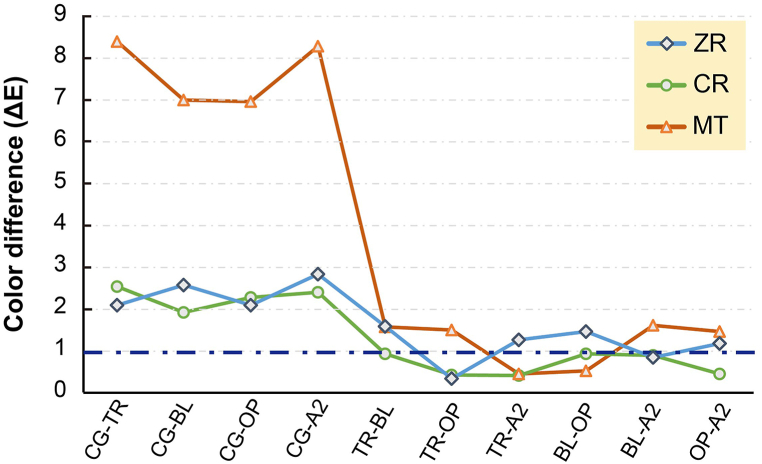
Comparison of the color difference of HT-Zr samples after adhering with cement of different colors under a specific substrate. Dotted line represents an acceptable color match of 1.0.

Fig. 4 shows the ΔE_00_ of various groups with the same substrate. The use of cement leads to a significant increase in ΔE_00_ regardless of the substrate (P < 0.05). In addition, only the CR group shows ΔE_00_ lower than acceptable color match (ΔE_00_ ranges from 0.8 to 1.0) after the use of cement. In the ZR group, only the ΔE_00_ of TR-OP (0.34 ± 0.21) and BL-A2 (0.85 ± 0.24) are lower than acceptable color match. In the MT group, only the ΔE_00_ of TR-A2 (0.45 ± 0.27) and BL-OP (0.53 ± 0.12) are lower than acceptable color match.

## 4 Discussion

The current study shows that the color differences produced when combining HT-Zr with different cement and substrates are significant; thus, the null hypothesis is rejected. This study used ZR, CR, and MT as substrates to simulate different abutments. The lightness (L* value) of substrates in descending order was ZR > CR > MT. As HT-Zr has high translucency (TP_00_ = 11.02 ± 0.18), its lightness is significantly affected by the L* of the substrate. Note that although MT shows low L*, the color difference is significant for HT-Zr on the MT (P < 0.05). For the same substrate, the lightness of the test groups in descending order is CG > BL > OP > A2 > TR. As the medium between HT-Zr and the test piece in the CG group is air, this group has the highest L*. The masking ability of TR cement is poor and tends to be affected by substrate color; thus, it has the lowest L*. BL, OP, and A2 cement all show masking effects. It should be noted that the difference in lightness is mainly due to the effect of the cement shade [28,29]. The C value in the MT group is significantly lower than that of other materials (P < 0.05). The greyer the substrate, the lower is the C value. As highly translucent HT-Zr and TR cement cannot effectively block the color of metals, the C value of this combination is the lowest (MT-TR = 3.95) [30].

Regardless of the substrate materials, the ΔE_00_ between the various groups and Vita A1 shade guide is higher when cement is used (Fig. 3). Light can directly penetrate and reflect substrate color in the CG group; however, the optical properties would be affected by the shade of both cement and substrates after applying cement. It was seen that if a significant color difference occurred before bonding, the future effect of cement on HT-Zr color change was limited, which is consistent with a previous study [27]. When excluding the CG group, only the CR group conforms to moderately unacceptable color match of ΔE_00_ = 3.6 (Fig. 3). Hence, when tooth color resin or natural tooth is used as an abutment material, HT-Zr exhibits good color accuracy regardless of the cement shade. The ΔE_00_ of the ZR group (from 4.46 to 5.44) is slightly higher than 3.6 (moderately unacceptable color match) but significantly more than the acceptable color match ranges (0.8–1.0; P < 0.05). When high-translucency zirconia or bleached tooth is applied as an abutment, staining is performed to adjust the final color of the restoration to achieve the expected color performance. The ΔE_00_ of the MT group (>11.88) is significantly higher than both acceptable and moderately unacceptable color match (P < 0.05). This is because the high translucency of HT-Zr remarkably reflects the metallic color of the substrate, so regardless of the masking effect of the cement, or even subsequent staining, the metal cannot be effectively masked [30]. Therefore, HT-Zr restorations may not be suitable for metal abutments or discolored teeth. While comparing the effect of different cement shade (Fig. 4), it was observed that the ΔE_00_ in all the CR groups (from 0.42 to 0.94) is lower than acceptable color match, and the ΔE_00_ of TR-A2 is the lowest (0.42 ± 0.20). It means that the effect of different cement shade on the ΔE_00_ of the CR group is weak. Thus, the difference in the final color of HT-Zr seems less noticeable, regardless if the cement shade matched with CR.

The current study has certain limitations, including the fact that only one shade and one type of HT-Zr was examined. The optical properties and morphology of HT-Zr after sandblasting, glazing, or polishing may be different. Although these limitations may affect the color accuracy of HT-Zr, the findings of this work have important implications that the abutment material could affect HT-Zr more than cement shade. When the difference between the color of the abutment and the color of the restoration is high, and the cement cannot completely mask the color of the abutment, the color accuracy of the restoration may not meet expectations. For patients with a natural tooth, if the abutment is discolored, stained, or bleached, it is recommended to perform masking for the abutment before restorations to achieve good color accuracy.

## 5 Conclusions

Within the limitations of this *in vitro* study, it can be concluded that when using HT-Zr the suitability of the abutment substrate materials must be considered in addition to the color of the cement to decrease color distortion and increase color accuracy for achieving the expected aesthetic performance. Additionally, the utilization of discolored or bleached abutments is not recommended for HT-Zr restorations.

## Data availability

Data will be made available on request.

## CRediT authorship contribution statement

**Chien-Ming Kang:** Writing - original draft, Conceptualization. **Yen-Wen Huang:** Funding acquisition, Formal analysis. **Sheng-Han Wu:** Investigation, Data curation. **Yuichi Mine:** Supervision. **I-Ta Lee:** Software. **Tzu-Yu Peng:** Writing - review & editing, Writing - original draft, Project administration, Conceptualization.

## Declaration of competing interest

The authors declare that they have no known competing financial interests or personal relationships that could have appeared to influence the work reported in this paper.
